# Resource allocation in the Covid-19 health crisis: are Covid-19 preventive measures consistent with the Rule of Rescue?

**DOI:** 10.1007/s11019-021-10045-0

**Published:** 2021-08-16

**Authors:** Julian W. März, Søren Holm, Michael Schlander

**Affiliations:** 1 Division of Health Economics, German Cancer Research Center (Deutsches Krebsforschungszentrum, DKFZ), Im Neuenheimer Feld 280, 69120 Heidelberg, Germany; 2 Centre for Social Ethics and Policy, Department of Law, University of Manchester, Manchester, UK; 3 Center for Medical Ethics, HELSAM, Faculty of Medicine, University of Oslo, Oslo, Norway; 4 Mannheim Medical Faculty, University of Heidelberg, Mannheim, Germany; 5 Institute for Innovation & Valuation in Health Care (InnoValHC), Wiesbaden, Germany

**Keywords:** Rule of Rescue, Covid-19 pandemic, Ethics of resource allocation, Resource allocation in healthcare

## Abstract

The Covid-19 pandemic has led to a health crisis of a scale unprecedented in post-war Europe. In response, a large amount of healthcare resources have been redirected to Covid-19 preventive measures, for instance population-wide vaccination campaigns, large-scale SARS-CoV-2 testing, and the large-scale distribution of protective equipment (e.g., N95 respirators) to high-risk groups and hospitals and nursing homes. Despite the importance of these measures in epidemiological and economic terms, health economists and medical ethicists have been relatively silent about the ethical rationales underlying the large-scale allocation of healthcare resources to these measures. The present paper seeks to encourage this debate by demonstrating how the resource allocation to Covid-19 preventive measures can be understood through the paradigm of the Rule of Rescue, without claiming that the Rule of Rescue is the sole rationale of resource allocation in the Covid-19 pandemic.

## Introduction

Resource allocation has long been a key issue for health economists and medical ethicists. Especially since the 1970s, various attempts have been made to ensure fair and sensible healthcare resource allocation in the light of increasing healthcare costs and limited healthcare budgets. Against this background, the utilitarian-inspired concept of economic “efficiency” of mainstream health economics has become increasingly popular with scholars and political decision-makers. Since the 1970s, the number of economic evaluations of healthcare programs conducted has virtually doubled every five years, and regulators and legislators worldwide have become increasingly reliant on considerations of economic “efficiency” to inform their funding decisions (see Neumann et al. 2017a, b; Widrig 2015). In the last two decades, for instance, cost-effectiveness studies have been made integral parts of funding decisions in healthcare in many countries worldwide, including the UK, France, Australia, and Canada (see Neumann et al. 2017a, b).

Despite this increasing popularity of the paradigm of economic “efficiency” in healthcare resource allocation, health economists have provided little support for political decisions on resource allocation to Covid-19 preventive measures (see Schlander 2020). At first look, this relative silence of health economists is surprising, given the fact that disaster ethics has generally assumed a shift towards a more utilitarian allocation of healthcare resources in disaster situations like a pandemic (Mallia 2015; Wagner and Dahnke 2015; Satkoske et al. 2019). It can, however, be explained by three reasons: Firstly, even if robust economic evaluation was available, political decision-makers would likely consider it inappropriate to make “efficiency” the main guideline of preventive measures against an infectious disease which threatens the health of every single citizen and which has cost more than four million lives worldwide and has led to the death of between 1 and 3% of the populations of most European and American States (by July 14, 2021, according to data from the Johns Hopkins Coronavirus Resource Center). Secondly, utilitarian-inspired economic evaluation of Covid-19 preventive measures is heavily dependent on robust empirical knowledge about Covid-19 in general (e.g., its age- and morbidity-dependent case-fatality ratio) and the epidemiology of Covid-19 in particular (e.g., the impact of certain preventive measures on infection rates), which remain disputed even more than one year after the first Covid-19 waves in Western States (see Schlander 2020). Thirdly, the epidemiological setting changes fast and Covid-19 preventive measures are time-critical, meaning that robust and reliable economic evaluation cannot be produced in time for political decision-making.

Given these limitations, paradigms other than economic “efficiency” appear more suited to understand the allocative decisions taken in response to the Covid-19 pandemic. Amongst these alternative paradigms, the Rule of Rescue (see Box 1 for a definition), which has been important in the academic debate on derogations from the mantra of economic “efficiency” in cases of life-threatening and life-changing diseases since its introduction by Jonsen (1986), is of particular significance.

The decisions on resource allocation in the Covid-19 pandemic were motivated by complex and multi-facetted ethical considerations, and it is not the purpose of the present paper to condense these considerations into one ethical concept (which, of course, would be impossible). Furthermore, it is not the purpose of the present paper to engage in the debate about whether the allocative decisions with regard to Covid-19 preventive measures were normatively justified at the time when they were made, or whether it is possible now to provide an adequate normative justification. Rather, the present paper seeks to show that Rule of Rescue considerations are helpful in order to capture and describe the allocative decisions taken during the Covid-19 pandemic, and that Rule of Rescue considerations may to some extent explain some of the decisions. That is, it is plausible that the decision-makers have used Rule of Rescue because they believed such considerations had normative or social force.

To this purpose, we will proceed in two steps: In a first step, we will discuss the concept of the Rule of Rescue in general as it was theorized before the Covid-19 pandemic. In a second step, we will demonstrate that the preventive measures taken by policymakers to tackle the Covid-19 pandemic are consistent with the Rule of Rescue.

Box 1: Definition of the Rule of Rescue

“*In order to save people from imminent peril, societies incur high costs largely irrespective of the fact that many more lives could be saved under alternative uses of the resources. This practice is usually referred to by the expression ‘Rule of Rescue’*” (Lübbe 2019).

The concept of the Rule of Rescue thus primarily describes the practice to allocate high amounts of healthcare or other resources to “rescue cases” and to largely ignore “efficiency” considerations in these cases.

In contrast, the Duty of Easy Rescue, which was theorized mainly by Peter Singer (e.g. Singer 1972) and Julian Savulescu (e.g. Savulescu 2007; Porsdam Mann et al. 2016; Giubilini et al. 2018a; Giubilini et al. 2018b; Koplin et al. 2020) is a normative principle that requires “*individuals* […] *to benefit others, or to prevent harm to others, when doing so entails a small cost to them*” (Giubilini et al. 2018a). The Duty of Easy Rescue equally applies to collectives (Giubilini et al. 2018b), i.e., also to policy-making in healthcare resource allocation, but only covers “easy” “rescue cases” (whereas costs and difficulty are not considered in the Rule of Rescue logic).

## Main part

### Conceptualization of the Rule of Rescue before the Covid-19 pandemic

In this first part, we will provide an overview^1^ of the process of conceptualization of the Rule of Rescue from Jonsen (1986) to McKie and Richardson (2003) and we will discuss the three key issues that lay at the heart of the “pre-Covid-19” debate on the Rule of Rescue.

#### The conceptualization of the Rule of Rescue from Jonsen (1986) to McKie and Richardson (2003)

The notion of the Rule of Rescue was introduced by Albert R. Jonsen in his seminal 1986 paper “*Bentham in a Box*”. Abstracting from theoretical considerations, Jonsen analyzed the practice of health technology assessments (to which he contributed as an ethicist) in which he observed what he labelled the Rule of Rescue. Whilst decision-makers are well prepared to be guided by economic “efficiency” when deciding on life-improving medical interventions, the same does not hold true once “life-saving” or “life-sustaining” medical interventions are at stake. According to Jonsen, the human moral impulse to save lives in peril termed the Rule of Rescue prevents these “life-saving” or “life-sustaining” medical interventions from being refused on “efficiency” grounds alone (Jonsen 1986).

The concept of the Rule of Rescue was further elaborated by Hadorn (1991). In his 1991 paper “*Setting health care priorities in Oregon. Cost-effectiveness meets the Rule of Rescue*”, Hadorn tried to analyze the public outrage at the first prioritized list introduced under the Oregon Basic Health Services Act (1989) which tried to combine an expansion of the franchise of Oregon’s Medicaid system with the rationing and prioritization of Medicaid healthcare benefits according to their economic “efficiency”. According to Hadorn’s analysis, a key flaw of Oregon’s first prioritized list (and also of the subsequent second prioritized list) was its incompatibility with the Rule of Rescue which he conceived as the principle that “*people cannot stand idly by when an identified person’s life is visibly threatened if effective rescue measures are available*” (Hadorn 1991).

Hadorn thus introduced two new aspects to the concept of the Rule of Rescue: Firstly, he abstracted from Jonsen’s analysis of the decision practice of health technology assessments and anchored the Rule of Rescue in the “human psyche”. Secondly, he introduced the concept of “identifiability” of victims into the debate on the Rule of Rescue. This concept describes the fact that humans have a strong psychological preference to allocate resources to persons currently ill (“identifiable victims”) as opposed to (yet unknown) victims who are merely predicted by statistics (“statistical victims”) (see Daniels 2012; Żuradzki 2015; Frick 2015).

Subsequently, these two aspects introduced by Hadorn (1991) were taken up by McKie and Richardson (2003) who were the first to provide an enumeration of features typically associated with the Rule of Rescue. According to their analysis, the Rule of Rescue can be observed in decisions on healthcare resource allocation which disregard opportunity costs in a situation where identifiable individuals are in peril of imminent and avoidable death or serious harm in which failure to act would be shocking. Furthermore, McKie and Richardson (2003) added a new dimension to the debate on the normativity of the Rule of Rescue: Whilst primarily following Hadorn (1991), who considered the Rule of Rescue a description of reality anchored in human psychology, they précised that acts in accordance with the Rule of Rescue could also be justified normatively since

“there is considerable social value in reinforcing acts driven by compassion and sympathy, and/or a sense of moral duty, even if the cost-effectiveness of such acts is only an incidental consideration. It is almost certainly true that people obtain benefit from the belief that they are living in a caring and humane society, and that the observation of attempts to save life, whether heroic or more mundane, reinforces this.” (McKie and Richardson 2003)

#### The three key issues shaping the “pre-Covid-19” debate on the Rule of Rescue

In more recent years, the debate on the Rule of Rescue has mainly centered around three key issues which remained unresolved: the normative status and implications of the Rule of Rescue, the applicability of the Rule of Rescue to the macroallocation of medical resources, and the relationship between the Rule of Rescue and economic “efficiency”.

##### Normative implications of the Rule of Rescue

McKie and Richardson (2003), although promoting a descriptive conception of the Rule of Rescue, had argued that the Rule of Rescue could potentially serve as a (normative) justification for acts that deviate from the principle of economic “efficiency” in rescue cases, because the rule could be given a normative justification based on the social value of living in a society that acts in ways that are caring and compassionate. The Rule of Rescue would thus be a derived normative principle, or at the very least a normatively justified heuristic. This conclusion was heavily contested by Cookson et al. (2008) who argued that applying the Rule of Rescue at the policy level would mean allowing irrationality and emotional intuitiveness in the process of macroallocation of medical resources. In the same vein, Jecker (2013) argued that the Rule of Rescue is an intuitive impulse unfounded on ethical principles, which risks distracting healthcare resources to “hopeless causes” to the detriment of all other patients.

Other authors, including Sheehan (2007), Lübbe (2015; 2017; 2019), and Largent and Pearson (2012), are, however, more prepared to justify allocative decisions in line with the Rule of Rescue, and also seem prepared to recognize some normative obligations on policymakers resulting from the Rule of Rescue (on recent attempts to establish the Rule of Rescue as an independent normative principle see in detail Rulli and Millum 2016). Largent and Pearson (2012), however, contend that, although decision-making on healthcare resource allocation on the basis of rescue impulses is ethically justifiable, the opportunity costs of these decisions should receive consideration.

##### Macroallocation of medical resources and the Rule of Rescue

Following Jonsen (1986), who had introduced the Rule of Rescue to explain decisions on the funding of “life-saving” and “life-sustaining” medical interventions, Hadorn (1991) and McKie and Richardson (2003) also invoked the Rule of Rescue in the context of macroallocation of medical resources. This focus of the concept of the Rule of Rescue on macroallocation has been seriously criticized by several authors. Cookson et al. (2008) argue that impulses to rescue individuals cannot simply be transferred to the policy level which does not decide on resource allocation to individual cases. Building on this argument, Orr and Wolff (2015) contended that the distinction between “identifiable” and “statistical” victims, which is at the heart of the concept of the Rule of Rescue of Hadorn and McKie and Richardson, is pointless at the policy level for which all (future) patients are merely “statistical” victims. In this regard, McKie and Richardson (2003) admitted that the paradigm of “identifiable” vs. “statistical” victims could possibly lead to a misallocation of medical resources to the benefit of highly mediatized cases, but to the detriment of “invisible” patient groups (for a more comprehensive overview over the debate on “identified” vs. “statistical” victims see Daniels 2012).

In this context, it must be noted that visibility can arguably blur the distinction between “identifiable” and “statistical” victims. If, for instance, the first (or current) patients in a given cohort are perceived as “identified” by decision-makers and the public, then the whole cohort, including future unknown patients, may be conceptualized as “identifiable”. In the Covid-19 pandemic, this logic plausibly applies to future critically ill Covid-19 patients, who are perceived rather as “identifiable” than as “statistical” victims by decision-makers and the public due to the high visibility of past and current critically ill Covid-19 patients.

In contrast, an application of the Rule of Rescue to the macroallocation of medical resources is notably defended by Sheehan (2007), Lübbe (2015; 2017; 2019), and Largent and Pearson (2012). Sheehan (2007) argues that policymakers in healthcare can potentially have both agent-neutral obligations of “efficiency” towards the general public and agent-relative obligations of rescue towards critically ill patients which must be weighed against one another. Lübbe (2015, 2017, 2019) contends that the Rule of Rescue must necessarily also be applicable to the macroallocation of medical resources since the macroallocation decisions predetermine possibilities of microallocation by medical staff “at the bedside” (to which the Rule of Rescue seems to be undisputedly applicable). Largent and Pearson (2012), however, call for a balancing of the Rule of Rescue with opportunity cost considerations, meaning that decisions which come at high total costs for the healthcare system (e.g., because of the large number of potential beneficiaries) are in their view not ethically justifiable.

##### Relationship between the Rule of Rescue and utilitarian-inspired economic “efficiency”

A majority of authors contributing to the debate on the Rule of Rescue, most notably Jonsen (1986) and McKie and Richardson (2003), but also Cookson et al. (2008) and Orr and Wolff (2015), are at least in principle subscribing to a model of “efficiency”-based and consequentialist allocation of healthcare resources. In their writings, the Rule of Rescue is conceived as a complement of economic “efficiency” in healthcare resource allocation to reflect the decision practice with regard to “life-saving” or “life-sustaining” medical interventions, which tends to largely ignore “efficiency” considerations. In contrast, deontologists, who also employ the Rule of Rescue in order to criticize the concept of economic “efficiency”, notably include Lübbe (2015; 2017; 2019) and potentially also Sheehan (2007).

### Consistency of Covid-19 preventive measures with the Rule of Rescue

Based on the above, we are going to demonstrate in the following that the preventive measures taken by policymakers in response to the Covid-19 health crisis, for instance population-wide vaccination campaigns, large-scale SARS-CoV-2 testing, and the large-scale distribution of protective equipment to high-risk groups and hospitals and nursing homes, are consistent with the Rule of Rescue and that the Rule of Rescue therefore might have played a relevant role in the processes leading to the decisions.

#### Covid-19 preventive measures as rescue measures

Prior to the Covid-19 health crisis, preventive measures did not have an important place in the academic debate on the Rule of Rescue. When introducing the Rule of Rescue in 1986, Jonsen primarily had “life-saving” medical interventions which benefitted currently critically ill patients (e.g., organ transplantation) in mind. The same holds true for McKie and Richardson (2003), for whom the imminence of death or serious harm (e.g., in the cases of patients in cardiac arrest or transplant patients requiring a further heart or liver transplant), was a key feature of the Rule of Rescue.

However, not only curative interventions, but also preventive interventions can constitute urgently and imminently needed “life-saving” treatments.

In this sense, Lübbe (2019) has argued (already prior to the pandemic) that a situation of “immediate peril” of death does not only arise at the point in time when there is imminence of death or serious harm, but rather already at the last point in time at which an action can be taken to prevent death or serious harm:

“The immediacy which triggers our sense of urgency is, in other words, not the immediacy of the death of an identified person, but the immediacy of the last point in time where an action can be taken that has a chance of avoiding probable death for an identified person.”

As an example, Lübbe (2019) discusses the case of non-metastasized malignant melanoma: She contends that it would be insensitive and illogical to delay the application of the Rule of Rescue until the tumor has metastasized and the patient requires intensive care, causing higher costs and severely worsening outcomes.

The same reasoning can easily be applied to infections with SARS-CoV-2 in high-risk groups. Certain high-risk groups, for instance persons above the age of 85, have lethality rates of SARS-CoV-2 infections of significantly more than 25% (Levin et al. 2020; see also Signorelli and Odone 2020 and for a more detailed estimate of age-specific SARS-CoV-2 infection fatality rates O’Driscoll et al. 2021). The last point in time in which sensible rescue measures to protect persons belonging to these high-risk groups from death or serious harm from SARS-CoV-2 infections can be implemented is before these infections have occurred. In this sense, Covid-19 preventive measures constitute no less rescue measures than curative measures targeting critically ill patients.

#### Insignificance of considerations of economic “efficiency”

In the traditional understanding of the Rule of Rescue of Jonsen (1986), Hadorn (1991), and McKie and Richardson (2003), a key feature of the Rule of Rescue is the neglect of “efficiency” considerations by healthcare policymakers with regard to “life-saving” or “life-sustaining” medical interventions.

Policymakers can decide to ignore “efficiency” considerations with regard to specified “life-saving” or “life-sustaining” medical interventions (e.g., the artificial heart discussed by Jonsen 1986), but also with regard to specified diseases, or with regard to particular situations. Furthermore, ignorance of “efficiency” considerations can be either explicit or implicit.

Many Covid-19 preventive measures (e.g., large-scale vaccination campaigns) will likely be proven to have been economically “efficient” by health economists in the future. Nevertheless, the fact remains that the expertise of health economists and considerations of economic “efficiency” have had little impact on the decisions on the allocation of healthcare resources to these measures.

## Conclusion

As the present paper has argued, Covid-19 preventive measures, for instance population-wide vaccination campaigns, large-scale SARS-CoV-2 testing, and the large-scale distribution of protective equipment to high-risk groups and hospitals and nursing homes, have been consistent with the Rule of Rescue.

The present paper does not claim that the Rule of Rescue has been the only or even the main rationale of decision-making on Covid-19 preventive measures. Rather, it has shown that the Rule of Rescue can be a helpful instrument in studying and analyzing the decisions on healthcare resource allocation taken during the Covid-19 pandemic.

Thereby, the present paper wants to encourage academic debate about the role that the Rule of Rescue has and should have in healthcare resource allocation. Furthermore, it seeks to encourage academic debate about the rationales of allocative decisions in the Covid-19 pandemic. As the present paper has underlined, the Rule of Rescue is a helpful instrument in understanding allocative decisions in healthcare, but it is neither a conclusive nor an exclusive answer.

## Data Availability

Not applicable.

## Footnotes

1 A more comprehensive account of proposed definitions of the Rule of Rescue is provided by Charlton (2021).
